# Treatment of synthetic textile wastewater containing dye mixtures with microcosms

**DOI:** 10.1007/s11356-017-0633-7

**Published:** 2017-11-06

**Authors:** Dina A. Yaseen, Miklas Scholz

**Affiliations:** 1 Civil Engineering Research Group, School of Computing, Science and Engineering, The University of Salford, Newton Building, Greater Manchester, M5 4WT UK; 2 Division of Water Resources Engineering, Department of Building and Environmental Technology, Faculty of Engineering, Lund University, P.O. Box 118, 22100 Lund, Sweden; 3 Department of Civil Engineering Science, School of Civil Engineering and the Built Environment, University of Johannesburg, Kingsway Campus, PO Box 524, Aukland Park Johannesburg, 2006 South Africa

**Keywords:** Aromatic amine, Artificial fabric effluent, Biological treatment, Chemical oxygen demand removal, Dyes mixture, *Lemna minor* L., Mineralisation, Nutrient

## Abstract

The aim was to assess the ability of microcosms (laboratory-scale shallow ponds) as a post polishing stage for the remediation of artificial textile wastewater comprising two commercial dyes (basic red 46 (BR46) and reactive blue 198 (RB198)) as a mixture. The objectives were to evaluate the impact of *Lemna minor* L. (common duckweed) on the water quality outflows; the elimination of dye mixtures, organic matter, and nutrients; and the impact of synthetic textile wastewater comprising dye mixtures on the *L*. *minor* plant growth. Three mixtures were prepared providing a total dye concentration of 10 mg/l. Findings showed that the planted simulated ponds possess a significant (*p* < 0.05) potential for improving the outflow characteristics and eliminate dyes, ammonium-nitrogen (NH_4_-N), and nitrate-nitrogen (NO_3_-N) in all mixtures compared with the corresponding unplanted ponds. The removal of mixed dyes in planted ponds was mainly due to phyto-transformation and adsorption of BR46 with complete aromatic amine mineralisation. For ponds containing 2 mg/l of RB198 and 8 mg/l of BR46, removals were around 53%, which was significantly higher than those for other mixtures: 5 mg/l of RB198 and 5 mg/l of BR46 and 8 mg/l of RB198 and 2 mg/l of BR46 achieved only 41 and 26% removals, respectively. Dye mixtures stopped the growth of *L*. *minor*, and the presence of artificial wastewater reduced their development.

## Introduction

### Background

The wastewater generated from textile factories is linked to one of the main water pollution problems. It contains a mixture of different dyes, auxiliaries, additives, and additional chemicals that were added during textile production processes, causing serious environmental concerns. However, the main problematic pollutants from textile factories in the aquatic environment are dye mixtures.

The direct discharge of dyes in concentrations higher than 1 mg/l, treated or not, could increase community complaints and concerns. This is primarily due to the aesthetic problem linked to these dyes, especially for the non-acceptable colours of river water such as red or purple compared to more accepted colours such as green or blue. In addition, textile dyes in high concentrations inhibit sunlight penetration, respiration activities and consequently upsetting the biological and photosynthesis processes in the aquatic environment.

Furthermore, the presence of these dyes for a long time in watercourses leads to dye accumulation in fishes and other organisms. Some dyes decompose, and corresponding hazardous compounds may also have a toxic impact on the aquatic environment (Carmen and Daniela 2012). Moreover, azo dyes, which are widely used in textile manufacturing and their daughter products (aromatic amine), can cause allergies, dermatitis, skin irritation, carcinogenic and mutagenic actions as well as acute and chronic toxicity (Yaseen and Scholz 2017; Carmen and Daniela 2012). Therefore, the treatment of these textile effluents is necessary.

Recently, among different methods used for the treatment of wastewater containing dyes, biological remediation using shallow wetland systems planted with aquatic plants has been recommended (Yaseen and Scholz 2017). This promising strategy is sustainable, low in costs, effective and environmentally friendly. It depends on the interaction between the plants, water and microbes for pollutant removal. However, apart from Yaseen and Scholz (2017; 2016) and Uysal et al. (2014), most studies on treating dye effluents using planted wetland systems are limited and were only operated for a short time (Muthunarayanan et al. 2011; Sivakumar 2014) without full assessments of the system performances.

Textile dyes in effluents are present as a mixture of different dyes according to the factory schedule, and in different percentages, depending on their degree of fixation with the fabric (Carmen and Daniela 2012). Therefore, it is imperative to assess particularly the removal of dye mixtures, which contain dyes in different degradation levels and mixing ratios. Treatment performances of mixtures of dyes with ozonation (Wijannarong et al. 2013), electrochemical oxidation (Chatizisymeon et al. 2006), bacterial biodegradation (Kolekar et al. 2013), fungal degradation (Taha et al. 2014), biosorption (Guendouz et al. 2016) and phytoremediation using Araceae, Portulacaceae and Verbenaceae have been reported by Kagalkar et al. (2010), Khandare et al. (2011a) and Kabra et al. (2012; 2011), respectively.

### Rational and novelty

No previous studies focused on using *Lemna minor* in wetland systems for the treatment of dye mixtures. In addition, most authors concentrated on mixtures of different dyes, which have been successfully treated individually. No investigations on the removal of mixtures of dyes at different levels of degradation have been performed, previously. Furthermore, a literature survey also indicated limited attention (Yaseen and Scholz 2017, 2016; Keskinkan and Lugal Goksu 2007) toward operating systems for dyeing wastewater treatment as a polishing step. Such a step is the last purification stage for pollutants in a multi-stage treatment system, which deals with wastewater characterised by relatively low concentrations of contaminants. The importance of this stage has increased as secondary treatment systems of biological, chemical or physical nature are unable to remove dye contaminations completely, and therefore, a further step is required.

This study addresses a knowledge gap in this area by operating pond systems planted with *L*. *minor* for the treatment of mixtures of two textile dyes containing wastewater. Reactive blue 198 and basic red 46 at total concentrations of 10 mg/l were used as representative dyes for long-term experiments as a polishing step.

*L. minor* is a tiny free-floating macrophyte belonging to the Lemnaceae family, able to remove dyes, heavy metals and nutrients from diverse wastewaters (Yaseen et al. 2017). The main mechanisms of dye removal by plants are phytodegradation and phyto-transformation in addition to adsorption and/or accumulation processes on/or within plants. This work offers a sustainable solution using a promising strategy to mitigate water pollution problems particularly for developing countries, which suffer from dye effluents, especially when financial resources are limited and land costs are relatively cheap.

### Aim and objectives

The aim is to explore the potential of simulated pond systems as polishing steps for the handling of synthetic textile wastewater comprising mixtures of RB198 and BR 46. The related objectives were to (i) evaluate the inflow water quality; (ii) assess the impact of *L*. *minor* on the outflow water quality and treatment performance in terms of dye mixtures, organic matter and nutrients removal; (iii) compare the dye mixture removal with each other and (vi) assess the impact of synthetic textile wastewater with dye mixtures on the plant development.

## Materials and methodologies

### Dyes and synthetic textile wastewater

Two commercial azo dyes were used in this project: RB198 and BR46; their characteristics and structures are illustrated in Table 1. The stock solutions were a mixture of 5 g of each dye, which were separately dissolved into 1 L of deionised water. The solutions were kept in total darkness at 4 °C. The dyes were used without further purification after receiving them from the supplier Dystar UK Limited (Colne Side Business Park, Huddersfield, England, UK). These two dyes were mixed together as mentioned below.

**Table 1:** Characteristics of dyes used in this study

Colour index name	Reactive Blue 198	Basic Red 46
Molecular composition	C_41_H_30_Cl_4_N_14_Na_4_O_14_S_4_	C_18_H_21_N_6_
Molecular weight (g/mol)	1304.8	321.4
λ_max_ (nm)	625	530
Chemical class	Diazo/oxazine (anionic)	Monoazo (cationic)
Dye content (%)	50–80	70–80

*λ*_*max*_ wavelength at maximum absorption, *C* carbon, *H* hydrogen, *N* nitrogen, *Na* sodium, *O* oxygen, *S* sulphur, *Cl* chlorine

The composition and concentration (g/l) of the chemicals used for preparing the synthetic textile wastewater were as follows: sodium benzoate (C_6_H_5_COONa, 0.1071), sodium acetate (CH_3_COONa; 0.2049), ammonium nitrate (NH_4_NO_3_; 0.1761), sodium chloride (NaCl; 0.007), magnesium chloride hexahydrate (MgCl_2_.6H_2_O; 0.0034), calcium chloride dehydrate (CaCl_2_.2H_2_O; 0.004) and potassium phosphate dibasic trihydrate (K_2_HPO_4_·3H_2_O; 0.0367). This recipe has been chosen in this study among many reported recipes because it has been previously examined in a biological treatment experiment undertaken by Ong et al. (2010). However, they used vertical-flow constructed wetlands vegetated with reeds.

In this study, a diluted solution of 1 part of artificial wastewater to 24 parts of raw water was used to help *L*. *minor* to survive. All chemicals were of analytical grade and applied without any further purification. The supplier was Scientific Laboratory Supplies Limited (Wilford Industrial Estate, Ruddington Lane, Wilford, Nottingham, England, UK). The stock solution of synthetic wastewater was prepared in the laboratory at a temperature of about 25 °C by mixing all compounds with de-chlorinated boiled tap water and stirred magnetically with a heating plate at 150 rpm for 1 h to ensure that all chemicals are completely dissolved.

For feeding the pond systems, three mixtures were prepared by combining the solutions of each dye, according to the required concentrations as mentioned below, together with the solution of the artificial wastewater and de-chlorinated tap water. Each mixture had a total concentration of 10 mg/l. The initial (inflow) dye concentrations in each mixture were as follows: mixture one (2 mg/l of RB198 and 8 mg/l of BR46), mixture two (8 mg/l of RB198 and 2 mg/l of BR46) and mixture three (5 mg/l of RB198 and 5 mg/l of BR46).

### Experimental set-up

The experiment was operated between 14 Oct. 2016 and 27 June 2017 under controlled laboratory conditions using plastic bowels (pond microcosms) of 33 cm length, 25.5 cm width and 14 cm depth. The plants were firstly taken from a pond near Cowpe Reservoir (Cowpe Lodge, Cowpe, Rossendale, England, UK), which was not impacted by any source of textile effluents and carefully rinsed with tap water, then kept under laboratory conditions to grow until the experiment started.

**Fig. 1:**
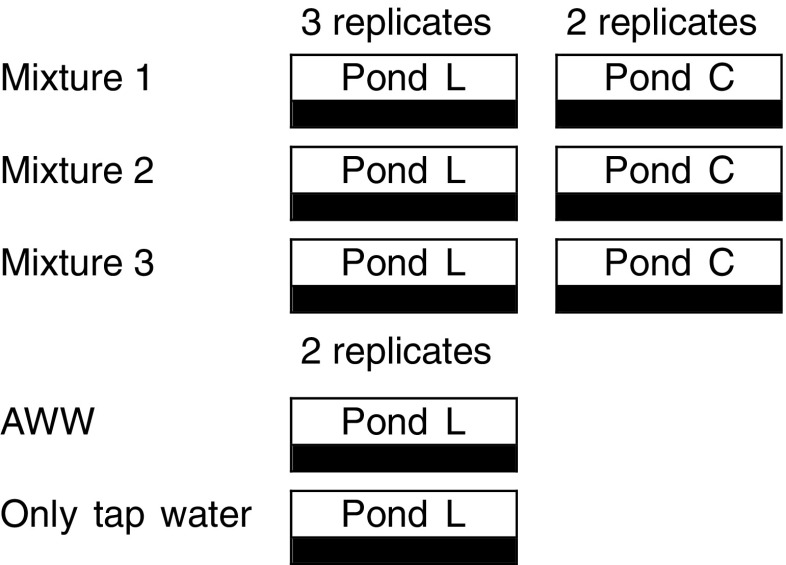
Overview of the experimental microcosm set-up. AWW artificial wastewater, Pond L *L*. *minor* L. pond, Pond C control pond

The experiment consists of 22 shallow pond simulations. Six ponds were used for each mixture of dyes and extra four containers had no dyes. The set-up for each mixture of dyes involved two treatment sets: one contained *L*. *minor* (Ponds L.; four replicates) and the other group had no plants as controls (Ponds C.; two replicates). The remaining four ponds were as follows: two planted ponds receiving artificial wastewater only without dye and two planted ponds receiving only tap water for comparison reasons. The set-up is shown in Fig. 1. All pond systems were filled with prepared wastewater up to a depth of 6.9 cm, which was equal to 5 l on 18 Oct. 2016 as a first dose. Subsequently, 200 healthy *L*. *minor* plants (about 2.6 ± 0.03 g) with approximately equal fond numbers were introduced to each system.

After adding the first dose, the system was fed weekly by removing the water solution in each pond (manually using suction pump) until the level was equivalent to 3 l (4.4 cm depth). Two additional litres were supplied to keep the water level equivalent to 5 l again. This is because the depth equivalent to 3 l was enough to keep the roots of *L*. *minor* without any contact to the base of the pond and to mimic the natural systems when the treated water was discharged and new doses were added. The retention time used was 7 days, which was similar to those times mentioned previously for the treatment of azo dyes using wetlands vegetated with *L*. *minor* (Yaseen and Scholz 2017, 2016). In addition, increasing the contact time consequently elevated dye elimination by *L*. *minor* (Reema et al. 2011).

### Analysis of samples

Regular samples of 50 ml were withdrawn for subsequent water quality assessments (APHA 2012) to monitor system performances. The spectrophotometer DR 2800 Hach Lange (Hach Lange, Willstätter Straße, Düsseldorf, Germany) was used to determine chemical oxygen demand (COD), absorbance, apparent colour and suspended solids (SS). Turbidity was obtained with a Turbicheck turbidity meter (Tintometer, Lovibond Water Testing, Dortmund, Germany). Both redox potential (redox) and pH were determined with a WTW Vario meter (Cole-Parmer Instrument Co., River Brent Business Park, Trumpers Way, Hanwell, London, England, UK). Dissolved oxygen (DO) was measured with a Hach HQ30d flexi meter (Hach, Pacific Way, Salford, England, UK). Both electric conductivity (EC) and total dissolved solids (TDS) were obtained with a METTLER TOLEDO Five Go™ meter (Keison Products, Chelmsford, England, UK).

Dye mixture assessments, which were applied according to the absorbance values, were performed after filtering the water samples (12 ml each) using a 0.45-μm diameter Whatman filter paper (Scientific Laboratory Suppliers Limited, Wilford Industrial Estate, Nottingham, UK). The filtered liquid was subsequently analysed with a UV–Vis spectrophotometer (DR 2800 Hach Lange) having a range between 400 and 800 nm at the maximum absorption wavelengths for each mixture of dye as discussed by Tony et al. (2009) for the measurement of the absorbance concerning the mixed dye as a new dye or new solution. However, the concentration measurements of each single dye before the combination were determined depending on standard calibration curves, which were computed for each dye by plotting the linear correlation line between known concentrations (mg/l) versus the absorbance at maximum absorption wavelengths for each dye, which was used to prepare the inflow mixtures. The maximum absorption wavelengths for each mixture were determined by scanning the aqueous solution using a Varian Cary 300 UV–Vis spectrophotometer with a range between 200 and 800 nm (www.varianinc.com). The corresponding wavelengths and absorbances were 528 nm and 0.449, 524 nm and 0.219 and 524 nm and 0.322 for the first, second and third mixtures, respectively.

High performance liquid chromatography (HPLC) as a biotransformation analysis was used for confirming organic molecule (dyes and their metabolites) separation by monitoring the peak area to find out, if it disappeared or shifted toward lower retention time. The tests were applied using Agilent 1260 on a HiChrom excel C18 column (4.3 to 250 mm; 1.7 μm particles). Acetonitrile (70%) and water (30%) were used at a flow rate of 1.3 l/min. The UV detector was kept at the maximum wavelength for each mixture. Aqueous samples of 20 μl were injected manually into the injector port using a microliter syringe.

A gas chromatography-mass spectrometry (GC-MS) test was conducted after sending the aqueous samples (1 l each in glass bottles) to Concept Life Sciences Analytical and Development Services Limited (Concept Life Sciences, Hadfield House, Hadfield Street, Cornbrook, Manchester, England, UK) for external analysis. Furthermore, nutrient and trace element analyses were applied (EPA 1994) for liquid samples using a Varian 720-ES Inductively Coupled Plasma-Optical Emission Spectrometer (Agilent Technologies Wokingham, Berkshire, England, UK). All samples (15 ml each) were cleaned of fines applying a Whatman filter paper (diameter; 0.45-μm), subsequently acidified using nitric acid S.G. 1.42 (> 68%) (Scientific Laboratory Suppliers Limited) and preserved at 4 °C.

Element contents within plant tissues were analysed as mentioned by Plank (1992) by using a Varian 720-ES Inductively Coupled Plasma-Optical Emission Spectrometer. The plants (both fresh and dried ones located on the pond sides) were harvested when the experiment was finished and dried in the oven at105 °C for 1 day (Sekomo et al. 2012), then grinded and sieved through a 2-mm diameter sieve. After that, around 0.2 g of each sample was added to a microwave tube with 10 ml of nitric acid. The samples plus an extra blank without plants were digested in the microwave (CEM Mars Xpress Microwave Digestion Oven) for 90 min until they were cooled-down. The samples were purified applying a Whatman filter paper (diameter of 0.45 μm) and diluted by deionised water up to volumes of 25 ml into volumetric flasks. The stock digest solutions were stored at 4 °C. Finally, further dilutions were conducted by adding 2.5 ml of the stock digest samples to 7.5 ml of deionised water in 12-ml centrifugal tubes, making in total four dilutions of the stored digest solution that is ready for analyses. Furthermore, the capacity of *L*. *minor* for phytoremediation of heavy metals (zinc and iron) was calculated according to the bioaccumulation factor, which is the ratio of element concentration in plant tissue (mg/kg) to the inflow concentration of each element (mg/l) as mentioned by Hegazy et al. (2011).

### Analysis of data

The standard software Microsoft Excel (www.microsoft.com) was used for data analysis. The IBM SPSS Statistics Version 22 (www.ibm.com) was applied to calculate the non-parametric Kruskal–Wallis and Mann-Whitney U tests for the non-normally distributed variables. The parametric *t* test was used for computing the normally dependent variables. The Shapiro-Wilk test was performed to assess, if data were normally distributed. The Spearman test was applied to determine the correlation coefficients of non-parametric parameters.

### Plant growth monitoring

The growth of *L*. *minor* was monitored to assess the impact of dye mixtures and artificial wastewater on plants. The fresh weights of *L*. *minor* used during the system set-ups were recorded. In addition, the remaining plants at the end of the experiment were harvested and the fresh weights were also recorded. This was undertaken after putting them on absorbent paper for 5 min. The corresponding dry weights were also noted after placing the harvested plants within an oven (105 °C; 1 day). The relative growth rate was calculated according to the fresh biomass as mentioned previously (Yaseen et al. 2017; Radic et al. 2010).

### Environmental monitoring

Budmaster Osram Delux (OD) led lamps (204 W) were used as grow lights. They were supplied by Budmaster LED (Unit 4, QHEP, Glan y Wern Road, Colwyn Bay, Gwynedd, Wales, UK). A timer controlled the light to simulate daylight in Salford (Time and Date 2016). The relative humidity and temperature readings were determined with the Thermometer-Hygrometer-Station provided by wetterladen24.de (JM Handelspunkt, Geschwend, Germany). Recordings of light were undertaken by applying the lux meterATP-DT-1300 between 200 and 50,000 lx (TIM-STAR, Road Three, Winsford Industrial Estate, Winsford, England, UK). Measurements were obtained from above the plants. The environmental boundary conditions are summarised in Table 2.

**Table 2:** Overview of environmental boundary conditions in the laboratory

Parameter	Unit	Mean	Standard deviation	Minimum	Maximum	Number
Temperature	°C	23.3	1.83	19.4	26.8	172
Temperature (minimum within 24 h)	°C	21.8	1.42	17.4	26.9	172
Temperature (maximum within 24 h)	°C	23.6	3.00	18.1	26.9	172
Relative humidity	%	64.0	4.05	51.0	73.0	172
Relative humidity (minimum within 24 h)	%	61.9	4.49	44.0	70.0	172
Relative humidity (maximum within 24 h)	%	69.9	5.39	53.0	80.0	172
Illuminance (one-off records)	lux	6853.5	382.9	6335	7722	96

## Results and discussion

### Overview of the inflow water quality

**Fig. 2:**
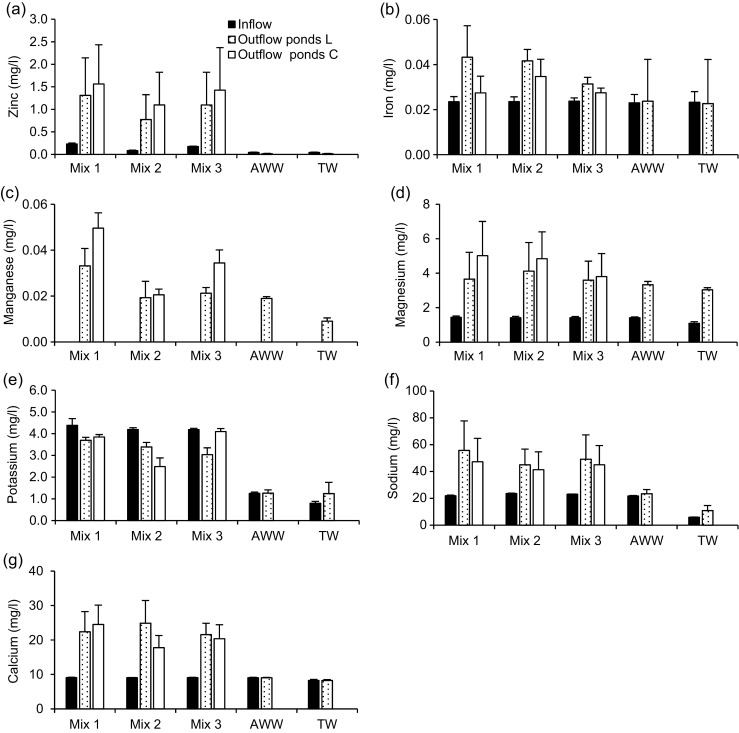
Overview of the average and standard deviations of the inflow and outflow values for the detected trace elements and heavy metals during the experiment. **a** Zinc. **b** Iron. **c** manganese. **d** Magnesium. **e** Potassium. **f** Sodium. **g** Calcium. Mix1, first mixture of 8 mg/l of basic red 46 and 2 mg/l reactive blue 198; Mix2, second mixture of 2 mg/l of basic red 46 and 8 mg/l of reactive blue 198; Mix3, third mixture of 5 mg/l of basic red 46 and 5 mg/l of reactive blue 198. AWW artificial wastewater, TW tap water, Pond L *Lemna minor* L. pond, Pond C control pond

The characteristics of the inflow water used in this study are summarised in Table 3. All mean inflow values were within the typical range of textile effluent characteristics (Ghaly et al. 2014), except for COD, SS and TDS concentrations, which were lower. The inflow dye concentrations of each mixture were 10.08 ± 0.35, 10.03 ± 1.01 and 10.04 ± 0.98 mg/l, respectively, which was within the lower range of typical concentrations of dye effluents (10–250 mg/l). The mean inflow concentrations of the elements detected through the ICP-OES analyses are presented in Fig. 2. The zinc and iron values were within the typical range of textile wastewater discharge of less than 10 mg/l (Ghaly et al. 2014). The inflow wastewater prepared and used in this study was suitable for operating pond systems as a polishing step (Yaseen and Scholz 2016).

**Table 3:** Inflow water quality parameters for the experiment between 18 October 2016 and 30 June 2017

Parameter	Unit	Mean	Standard deviation	Minimum	Maximum	Number
Mixture 1						
pH	–	7.3	0.11	7.1	7.5	34
Redox	mv	− 41.9	5.42	− 54.0	− 33.0	34
Dissolved oxygen	mg/l	8.9	0.30	8.3	9.4	34
Electronic conductivity	μS/cm	109.9	14.20	88.9	128.0	34
Total dissolved solids	mg/l	55.0	7.10	44.5	64.0	34
Suspended solids	mg/l	2.3	0.76	1.0	3.0	34
Turbidity	NTU	2.4	0.39	1.8	3.4	34
Colour	Pt Co	571.4	47.06	452.0	615.0	34
Absorbance	–	0.45	0.016	0.43	0.520	34
Chemical oxygen demand	mg/l	33.7	0.30	33.0	34.0	9
Ammonia-nitrogen	mg/l	0.24	0.021	0.22	0.288	9
Nitrate-nitrogen	mg/l	0.58	0.027	0.52	0.610	9
Ortho-phosphate-phosphorus	mg/l	1.71	0.119	1.50	1.940	9
Mixture 2						
pH	–	7.2	0.09	7.1	7.4	34
Redox	mv	− 41.8	5.70	− 50.0	− 32.0	34
Dissolved oxygen	mg/l	8.8	0.29	8.2	9.3	34
Electronic conductivity	μS/cm	112.4	15.47	85.5	131.9	34
Total dissolved solids	mg/l	56.2	7.73	42.8	66.0	34
Suspended solids	mg/l	3.0	1.22	2.0	5.0	34
Turbidity	NTU	3.1	0.56	2.1	3.6	34
Colour	Pt Co	254.7	17.76	220.0	288.0	34
Absorbance	–	0.22	0.022	0.12	0.251	34
Chemical oxygen demand	mg/l	29.2	0.35	28.8	30.0	9
Ammonia-nitrogen	mg/l	0.19	0.013	0.17	0.22	9
Nitrate-nitrogen	mg/l	0.48	0.027	0.44	0.53	9
Ortho-phosphate-phosphorus	mg/l	1.59	0.052	1.48	1.65	9
Mixture 3						
pH	–	7.2	0.08	7.1	7.3	34
Redox	mv	− 39.3	4.77	− 46.0	− 32.0	34
Dissolved oxygen	mg/l	8.9	0.37	8.2	9.5	34
Electronic conductivity	μS/cm	112.1	15.54	86.6	132.1	34
Total dissolved solids	mg/l	56.1	7.77	43.3	66.1	34
Suspended solids	mg/l	2.8	0.86	2.0	4.0	34
Turbidity	NTU	3.0	0.57	2.1	3.6	34
Colour	Pt Co	411.5	37.33	338.0	469.0	34
Absorbance	–	0.32	0.032	0.21	0.391	34
Chemical oxygen demand	mg/l	31.8	0.35	31.2	32.5	9
Ammonia-nitrogen	mg/l	0.22	0.022	0.17	0.260	9
Nitrate-nitrogen	mg/l	0.53	0.025	0.49	0.580	9
Ortho-phosphate-phosphorus	mg/l	1.64	0.028	1.60	1.700	9
Artificial wastewater						
pH	–	7.1	0.07	7.0	7.3	31
Redox	mv	− 26.2	7.16	− 37.0	− 18.0	34
Dissolved oxygen	mg/l	9.2	0.42	8.5	9.7	34
Electronic conductivity	μS/cm	89.9	1.34	88.5	95.4	34
Total dissolved solids	mg/l	45.0	0.67	44.3	47.7	34
Suspended solids	mg/l	1.5	0.50	1.0	2.0	34
Turbidity	NTU	1.6	0.43	1.0	2.1	34
Colour	Pt Co	8.0	1.71	6.0	12.0	34
Chemical oxygen demand	mg/l	18.5	0.16	18.1	18.7	9
Ammonia-nitrogen	mg/l	0.16	0.004	0.16	0.17	9
Nitrate-nitrogen	mg/l	0.41	0.020	0.38	0.44	9
Ortho-phosphate-phosphorus	mg/l	1.19	0.038	1.11	1.25	9

*NTU* nephelometric turbidity unit

### Outflow water quality parameters

#### Dye and apparent colour

**Fig. 3:**
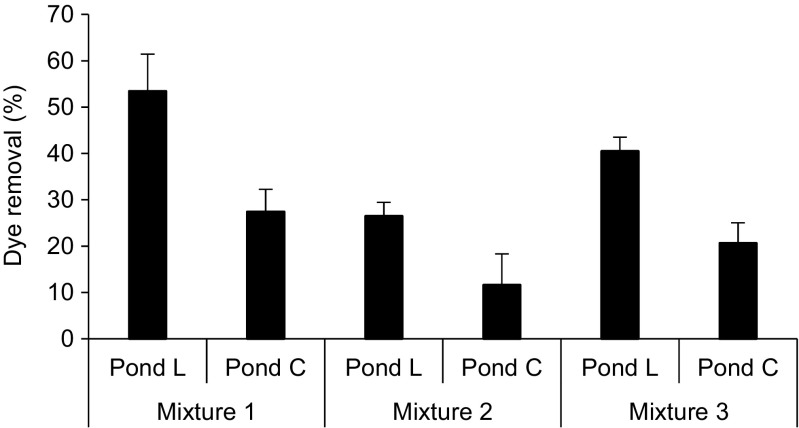
Overview of the mean removal efficiencies of dye mixtures during the experiment period between 14 October 2016 and 27 June 2017. Pond L *Lemna minor* L. pond, Pond C control pond. Mixture 1, 8 mg/l of basic red 46 and 2 mg/l reactive blue 198; mixture 2, 2 mg/l of basic red 46 and 8 mg/l of reactive blue 198; mixture 3, 5 mg/l of basic red 46 and 5 mg/l of reactive blue 198. Note that error bars indicate the standard deviations among the removal values

Findings showed that the mean removal efficiency of each mixture in ponds planted with *L*. *minor* was significantly (Mann-Whitney U, *p* < 0.001) higher than unplanted ponds (Fig. 3). This indicates the high influence of *L*. *minor* by enhancing the potential of dye removal within the ponds. The mean removal values of dye mixtures were as follows: mixture one > mixture three > mixture two for both planted and control ponds. Significant differences were found among all planted ponds (Kruskal–Wallis , *p* < 0.05). However, control ponds showed that the removal values of mixture two were significantly lower than the corresponding removals of mixtures one and three (Kruskal–Wallis , *p* < 0.05).

The results of mixed dye elimination attributed to the impact of dye BR46 removal in each solution. This is because BR46 exhibited a high percentage of degradation by *L*. *minor* ponds of around 85% in short-term studies and 64% in long-term studies at concentrations of 10 mg/l (data not shown) and 69% at concentration of 5 mg/l compared with very low or negligible removal linked to the dye RB198 (Yaseen and Scholz 2016). Therefore, mixture one, which contained 80% of BR46, displayed higher removal than mixture three, which had 50% of BR46, whereas lower removal was found in mixture two comprising only 20% of BR46. This suggests that the removal levels by pond systems are stable. However, high loads of BR46 led to achieving high percentages of degradation. This observation matched the findings by Davies et al. (2006).

The authors noticed that although the removal rate of the system was constant, the percentage of dye degradation enhanced when the loading rate of dyes was rising. By comparing the mean removal of dye mixtures achieved in this study (Fig. 3) with the individual dye BR46 removal values as mentioned above, the results clearly showed that RB198, which is recalcitrant to degrade, had an adverse impact on the removal efficiency of the mixture. The inflow and outflow absorbance values (Tables 3 and 4) reflect the mixed dye elimination findings.

**Table 4:** Overview of the mean outflows water quality parameters for the experiment between 18 October 2016 and 30 June 2017

Parameter	Unit	Mean	Standard deviation	Mean	Standard deviation	Number
Mixture 1		*Lemna minor* L. ponds		Control ponds		
pH	–	7.4	0.12	7.5	0.17	34
Redox	mv	− 54.9	7.10	− 58.5	10.61	34
Dissolved oxygen	mg/l	8.5	0.18	8.5	0.17	34
Electronic conductivity	μS/cm	115.8	11.99	117.5	17.89	34
Total dissolved solids	mg/l	57.9	6.00	58.8	8.95	34
Suspended solids	mg/l	5.1	1.38	3.2	1.82	34
Turbidity	NTU	4.3	1.49	3.4	0.38	34
Colour	Pt Co	277.0	81.66	447.6	124.00	34
Absorbance	–	0.14	0.045	0.29	0.057	34
Chemical oxygen demand	mg/l	12.13	2.232	13.10	3.506	9
Ammonia-nitrogen	mg/l	0.06	0.027	0.10	0.041	9
Nitrate-nitrogen	mg/l	0.21	0.023	0.32	0.089	9
Ortho-phosphate-phosphorus	mg/l	2.00	0.373	2.41	0.455	9
Mixture 2		*L*. *minor* ponds		Control ponds		
pH	–	7.5	0.13	7.5	0.11	34
Redox	mv	− 54.9	7.58	− 56.1	6.26	34
Dissolved oxygen	mg/l	8.5	0.14	8.5	0.14	34
Electronic conductivity	μS/cm	114.8	14.57	121.5	13.71	34
Total dissolved solids	mg/l	57.4	7.29	60.8	6.86	34
Suspended solids	mg/l	5.3	1.74	3.4	1.09	34
Turbidity	NTU	4.9	1.75	3.3	0.45	34
Colour	Pt Co	151.0	26.85	210.9	46.68	34
Absorbance	–	0.13	0.020	0.21	0.043	34
Chemical oxygen demand	mg/l	10.80	1.995	11.40	2.458	9
Ammonia-nitrogen	mg/l	0.06	0.014	0.13	0.064	9
Nitrate-nitrogen	mg/l	0.16	0.029	0.24	0.055	9
Ortho-phosphate-phosphorus	mg/l	1.62	0.249	2.16	0.309	9
Mixture 3		*L*. *minor* ponds		Control ponds		
pH	–	7.4	0.14	7.5	0.12	34
Redox	mv	− 54.6	7.68	− 57.4	7.10	34
Dissolved oxygen	mg/l	8.4	0.14	8.5	0.15	34
Electronic conductivity	μS/cm	115.1	13.12	119.4	12.85	34
Total dissolved solids	mg/l	57.5	6.56	59.7	6.43	34
Suspended solids	mg/l	4.4	1.69	3.3	1.16	34
Turbidity	NTU	4.1	1.22	3.3	0.39	34
Colour	Pt Co	199.7	49.28	327.7	80.11	34
Absorbance	–	0.14	0.023	0.23	0.031	34
Chemical oxygen demand	mg/l	11.66	3.378	12.19	3.208	9
Ammonia-nitrogen	mg/l	0.05	0.014	0.09	0.058	9
Nitrate-nitrogen	mg/l	0.13	0.043	0.21	0.062	9
Ortho-phosphate-phosphorus	mg/l	1.78	0.309	2.19	0.388	9
		Artificial wastewater and *L*. *minor* ponds		Tap water. *L*. *minor* ponds		
pH	–	7.5	0.14	7.4	0.13	34
Redox	mv	− 60.3	8.87	− 54.4	7.14	34
Dissolved oxygen	mg/l	8.3	0.76	8.4	0.16	34
Electronic conductivity	μS/cm	110.6	14.98	93.1	8.98	34
Total dissolved solids	mg/l	55.3	7.49	46.6	4.49	34
Suspended solids	mg/l	3.9	2.50	3.0	1.71	34
Turbidity	NTU	3.8	0.83	3.5	0.68	34
Colour	Pt Co	32.0	17.86	27.1	11.27	34
Chemical oxygen demand	mg/l	9.60	1.043	4.74	0.840	9
Ammonia-nitrogen	mg/l	0.04	0.018	0.02	0.004	9
Nitrate-nitrogen	mg/l	0.06	0.075	0.05	0.049	9
Ortho-phosphate-phosphorus	mg/l	0.87	0.403	0.40	0.289	9

*NTU* nephelometric turbidity unit

**Fig. 4:**
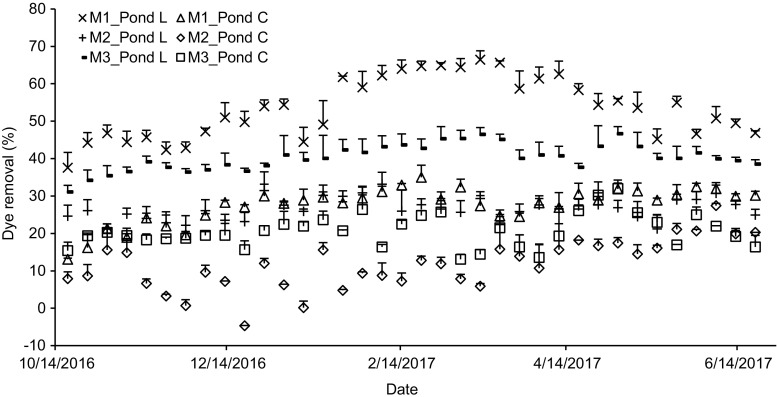
Mean values for dye removal profile. Pond L *Lemna minor* L. pond, Pond C control pond. M1, 8 mg/l of basic red 46 and 2 mg/l reactive blue 198; M2, 2 mg/l of basic red 46 and 8 mg/l of reactive blue 198; M3, 5 mg/l of basic red 46 and 5 mg/l of reactive blue 198. Note that error bars indicate the standard deviations for the replicates

The longitudinal profiles and trends of the mixed dye removals are shown in Fig. 4, highlighting that the removal values were low and fluctuated during the first period of the experiment. This may attribute to consider the first period as an acclimatisation stage for the plants and organisms within the ponds. The inflows contained mixtures of two dyes and artificial wastewater chemicals.

The amount of plants was limited according to the set-up design. Plants may require enough time to grow and regenerate due to the shock received from the dye mixture dosages. However, higher removal was achieved in planted ponds between 17 January 2017 and 18 April 2017, which is possible, because the biomass of *L*. *minor* reached a high level. Then, the plants started to become saturated with dyes, except for the plants within mixture two. This consequently led to a noticeable reduction in the removal of planted ponds, especially ponds treating mixture one. The maximum and minimum mean removal values were as follows: for ponds treating mixture one, 66 and 38% for *L*. *minor* ponds and 35 and 13% for control ponds; for ponds comprising mixture two, 33 and 20% for planted ponds and 27 and − 5% for control ponds, respectively and for ponds containing mixture three, 47 and 31% for planted ponds and 32 and 13% for unplanted ponds in that order. Negative removal values were found in control ponds treating mixture two, which contained a high percentage of RB198. This was attributed to dried RB198 continuously attaching itself to the walls of the pond sides (Yaseen and Scholz 2016).

The findings of dye mixture removal confirmed the low removal of RB198 and high degradation of BR46 that has previously been reported by Yaseen and Scholz (2016). The main cause of high BR46 removal are the absence of sulphonic groups, low molecular weight and the simple chemical structure compared to RB198 (Yaseen and Scholz 2017).

Regarding apparent colour monitoring (Table 4), the average outflow colour numbers for both planted and control ponds were lower than the corresponding inflow numbers (Table 3) for all mixtures. In addition, all outflow colour values in the planted ponds were highly significantly lower (Mann-Whitney U, *p* < 0.001) than the unplanted ones. This reflects the significant removal achieved for dye mixtures in planted ponds compared with the control ones, which consequently reduced the apparent colour in the planted system. The colour of inflow samples was dark red, purple and red for mixtures one, two and three, respectively. However, the outflow samples were rather colourless for planted ponds and light pink for control ponds containing mixture one. Blue and dark blue outflows were found in planted and control ponds treating mixture two, and, finally, blue and light purple outflow was recorded for planted and unplanted ponds treating mixture three. The colour parameter of ponds without dyes showed a significantly (Kruskal–Wallis , *p* < 0.05) reduced outflow colour compared to ponds containing dyes.

#### Monitoring of ultraviolet spectral changes

**Fig. 5:**
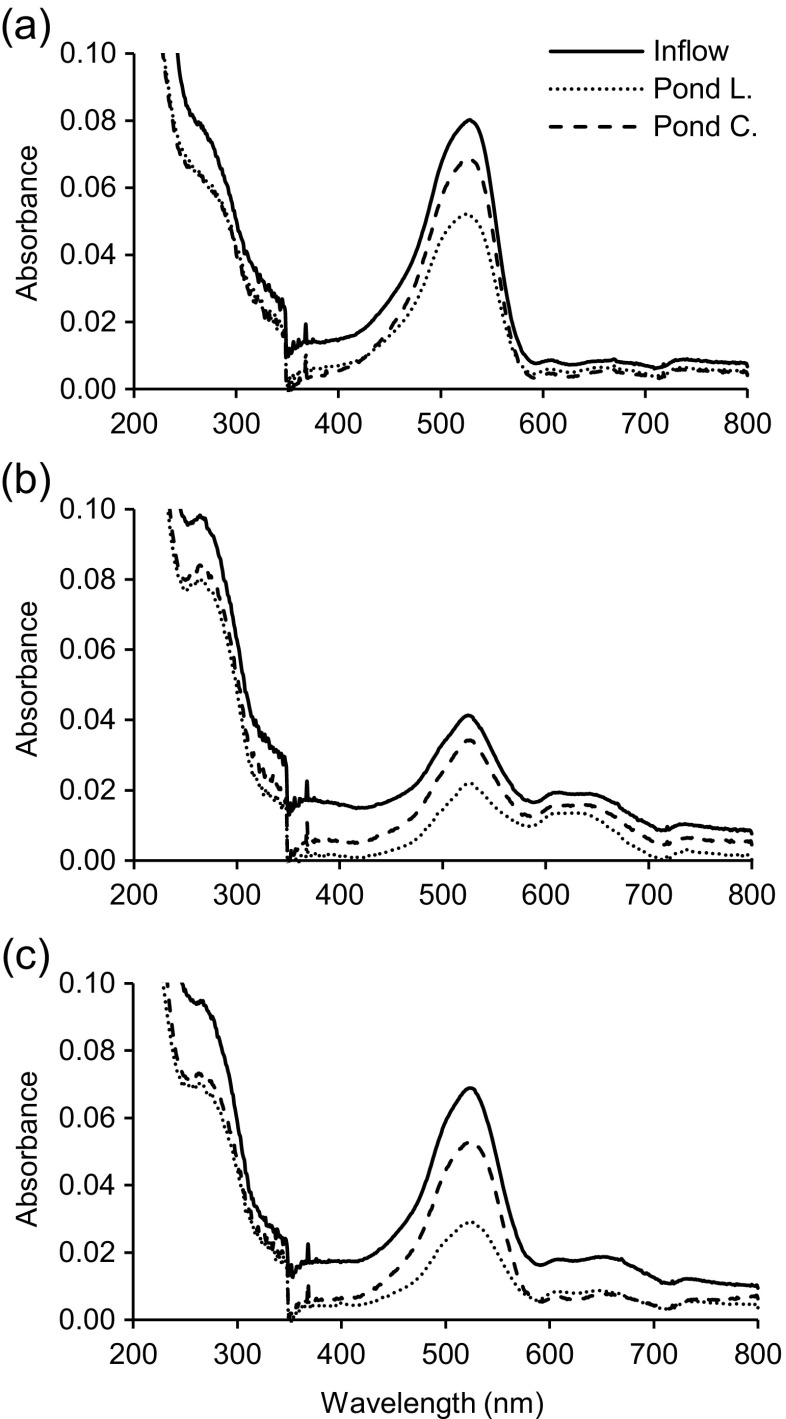
Ultraviolet visible spectra for each mixture before and after treatment. **a** Mixture 1 (8 mg/l of basic red 46 and 2 mg/l reactive blue 198). **b** Mixture 2 (2 mg/l of basic red 46 and 8 mg/l of reactive blue 198). **c** Mixture 3 (5 mg/l of basic red 46 and 5 mg/l of reactive blue 198). Pond L outflow of ponds planted with *Lemna minor* L., Pond C outflow of control ponds

Ultraviolet visible scans showed that the maximum adsorption wavelength of each separate dye was 625 and 530 nm for RB198 and BR46, respectively (Table 1). However, the inflow mixtures, after dilution, showed maximum absorbances at wavelengths of 528, 524 and 524 nm with dark red, purple and red inflow colours for mixtures one, two and three, respectively (Fig. 5). This was due to the interference between dyes mixed together. The outflow samples illustrated a reduction of the dye intensity for unplanted ponds, which was due to dye adsorption by microbes in the system. The intensity dropped in planted ponds for all mixtures (Fig. 5). This confirmed the higher decolourisation of each mixture in planted ponds compared to the control ones. The peaks for the outflow samples in this study did not completely disappear, which indicated that particularly, dye RB198 was difficult to biologically degrade.

Extra peaks appeared in the decolourised samples at 282, 277 and 277 nm for mixtures one, two and three in this order. These new peaks probably resulted from the metabolites or degradation of dye BR46. Similar results and explanations have been reported by Chen et al. (2003).

#### Chromatographic analysis

The HPLC chromatogram analysis for dye mixtures before treatment showed two minor peaks besides the main peak at 3.385 min for mixture one. However, the outflow samples for planted ponds showed that the main peak disappeared and new major peaks appeared at 1.88 and 2.58 min besides several minor peaks. On the other hand, the control ponds showed only appearances of new major peaks at 1.543 and 2.101 min. Regarding the second mixture, the inflow samples presented major peaks at 2.175 min with minor peaks at 1.49 and 1.9 min. The outflow samples of planted ponds showed that the inflow peaks disappeared with appearances of major peaks at 2.175 min in addition to several minor peaks. In comparison, the control ponds had major peaks at 2.155 min and minor peaks at 2.714 and 2.37 min. Finally, the third mixture showed major peaks for inflow samples at 2.813 min and two other minor peaks. However, the outflow samples for planted ponds were linked to a major peak at 1.7 min and a minor one at 2.216 min. The control ponds had a major peak at 2.207 min and other minor ones (data not shown).

The variation between the inflow and outflow samples concerning the presence of new peaks can be explained by the formation of different products during molecule transformation and consequently decolourisation (Joshi et al. 2010; Kalyani et al. 2009). These changes occurred for both planted and control ponds due to dye BR46 degradation, although the mechanism and percentage of elimination was different. As a result, the mechanism of removal was due to phyto-transformation with adsorption and/or accumulation by plants (Kabra et al. 2012; Khandare et al. 2011b) besides the microbial impact in planted ponds. However, the control pond potential was due to microbial biosorption.

The results of GC-MS analysis related to mixture one showed that the treated samples of *L*. *minor* ponds did not contain aromatic amines. This indicates that the cleavage products completely mineralised. Although aromatic amines are toxic to plants and organisms, Sponza and Isık (2005) confirmed that toxicity effects were eliminated during the decolourisation process being followed by complete mineralisation. However, the control ponds provide outflows containing one trace peak for N-(4-methylphenyl)-benzenemethanamine (CAS 5405-15-2). The concentration of this amine was below 10 mg/l, which was the detection limit of the machine. This aromatic compound was considered as non-hazardous material. However, as an environmental precaution, discharge of this compound should be avoided. Toxicological and ecological properties were not evaluated. Pond systems without plants were unable to achieve complete mineralisation.

#### Chemical oxygen demand and nutrients

**Fig. 6:**
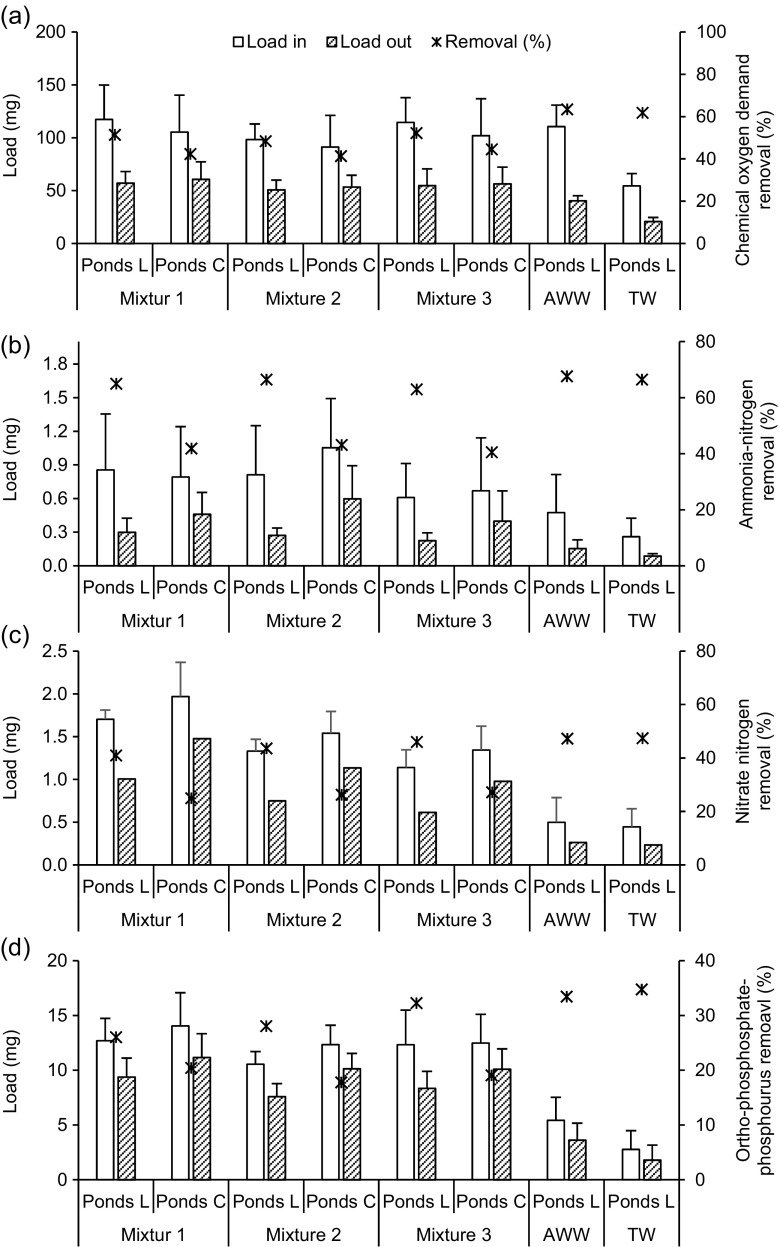
Overview of mean removal efficiencies during the experimental period. **a** Chemical oxygen demand. **b** Ammonium-nitrogen. **c** Nitrate-nitrogen. **d** Ortho-phosphate-phosphorus. Mixture 1, 8 mg/l of basic red 46 and 2 mg/l of reactive blue 198; mixture 2, 2 mg/l of basic red 46 and 8 mg/l of reactive blue 198; mixture 3, 5 mg/l of basic red 46 and 5 mg/l of reactive blue 198. AWW artificial wastewater, TW tap water, Pond L *Lemna minor* L. pond, Pond C control pond

The changes between COD concentrations before and after treatment confirmed degradation processes of organic matter in the system, and consequently dye molecule degradation. Tables 3 and 4 show that the mean COD values after treatment were less than the average inflows. Moreover, all mean outflow COD values in *L*. *minor*-planted wetlands were lower than the values for the control ponds. This has been confirmed by COD removal values (Fig. 6a), which were higher in planted ponds than the control ones. Consequently, dye degradation was better in planted than unplanted ponds. However, no significant (*t* test, *p* > 0.05) differences were found in case of COD mean outflow values and COD removal efficiencies. These results confirm the low impact of plants on COD degradation besides microbial activates. Similarly, a negligible impact of COD removal by reeds was concluded by Ong et al. (2009).

The removal of COD for all simulated ponds containing dyes was lower in comparison to those ponds without dyes (Fig. 6a). This may be attributed to the impact of incomplete organic molecule degradation for all dye mixtures. Comparable findings were reported by Sarayu et al. (2007) using ozonation for dye removal. The authors found that the presence of small molecules of untreated dyes has a considerable contribution to incomplete COD reduction. Common international standards state limits for COD of around 125 mg/l in case of direct discharge. The results highlight that all COD outflow concentrations were below this limit.

Based on nitrogen removal, the eutrophication that was caused by adding nitrogen to the surface water and the negative impacts on some aquatic organisms, which was caused by the presence of NH_4_-N in receiving watercourses, even in low concentrations, makes the removal of nitrogen a very important parameter for wastewater treatment systems. The main processes for nitrogen reduction in pond systems are sedimentation, nitrification and denitrification, as well as nutrient assimilation by biomass. Bragato et al. (2006) confirmed that wetland plants potentially sequester nutrients from wastewater to their roots and/or shoots, and, as a result, they remediate these pollutants due to the rapid growth and biomass production of these macrophytes.

Regarding NH_4_-N and NO_3_-N, all mean outflow values (Table 4) were lower, if compared with the corresponding inflow concentrations (Table 3), indicating a reduction of these two compounds in the system. Also, control ponds showed higher outflow concentrations than the corresponding values associated with *L*. *minor* ponds. This was attributed to the significant (*p* > 0.05) removal of NH_4_-N (*t* test) and NO_3_-N (Mann-Whitney U test) in planted ponds compared to the corresponding removal related to unplanted wetlands (Fig. 6b, c, respectively).

Ong et al. (2009) highlighted that the effect of vegetation on nitrogen reduction in wetland systems is not evident in all applications and depends on the type of plants, operational period and wastewater characteristics. However, the results of this study confirmed a considerable impact of the plants for take-up of nitrogen, which may ascribe to the low loading rate of nitrogen in the system. Similarly, Selvarani et al. (2015) elucidated that *L*. *minor* has a vital role for nitrogen reduction in pond systems. In addition to the plants, nitrification and denitrification processes are also responsible for NH_4_-N and NO_3_-N reductions, respectively. Temperature and pH values in addition to the high concentrations of oxygen in all ponds were suitable for enhancing the nitrification level by nitrifying bacteria (Kadlec et al. 2000; Ozengin and Elamic 2007).

Denitrification occurs within the anoxic or anaerobic zone below the mat of *L*. *minor*, which is located below the top water layer of the system (partly or fully) covered by *L*. *minor* and the anaerobic sediment on top of the bottom plastic container walls. Nitrogen reduction of around 4% can be attributed to nitrification and denitrification occurring on micro-sites of the biofilm attached to *L*. *minor* (Zimmo 2003). The mean reduction values of NO_3_-N were not as high as those for NH_4_-N, which may either be because of the plants using NO_3_-N after assimilation of ammonia as a second source for nutrients or because the environmental conditions within the ponds were not suitable for high denitrification to occur. In addition, the high level of nitrification linked with oxygen availability reflects the increase of NO_3_-N concentration in the pond systems (Vymazal 2007).

The typical international limits for NH_4_-N and NO_3_-N regarding secondary treatment of effluent are 20 and 50 mg/l in this order as discussed by Al-Isawi et al. (2017). All outflow values of NH_4_-N and NO_3_-N were less than the corresponding standard thresholds.

Concerning ortho-phosphate-phosphorus (PO_4_-P), higher mean outflow values (Table 4) were found, if compared to the corresponding inflow records (Table 3). Also, the mean outflow PO_4_-P concentrations were significantly lower (*t* test, *p* < 0.05) in *L*. *minor* ponds compared to the ponds without plants, reflecting better removal of PO_4_-P in ponds containing *L*. *minor* than the control ones, although the system showed low overall reductions for all ponds (Fig. 6d). This outcome resembles that of other authors: wetland systems are relatively ineffective in phosphorus removal (Vymazal 2007; Al-Isawi et al. 2017).

Significant removal (*t* test, *p* < 0.05) of PO_4_-P between planted and unplanted ponds was noticed in ponds treating mixtures two and three. This may be due to the state of *L*. *minor* plants in ponds containing mixture one, which were not well if compared to the other ponds, making their ability to assimilate phosphorus not effective. The expected mechanisms for PO_4_-P removal, except for chemical precipitation, were due to the uptake by plants, especially mixtures two and three, and microbes in planted ponds (Vanitha et al. 2013). However, only biological processes associated with microbial uptake were possible in control ponds. A common international limit for PO_4_-P outflow is 1 mg/l as indicated by Sani et al. (2013). In this study, all outflow values for ponds containing dyes were higher than this standard value.

#### Heavy metals and trace elements

The main source of elements in this study was the artificial wastewater and to a lesser extent some elements in tap water. In addition, zinc ions were present in the dye BR46. Although some of the elements are important for plants as micro-nutrients, these elements could be toxic at high concentrations. The plants play an important role in element phytoremediation due to active and/or passive transport of elements in wetlands (Bonanno and Vymazal 2017).

Mean outflow zinc and iron values (Fig. 2a, b, respectively) were higher than the corresponding inflow concentrations for both planted and control ponds. Ponds containing *L*. *minor* showed lower Zn outflows than the control ones reflecting zinc uptake by plants, although no significant (*p* > 0.05) differences were found for all mixtures. All zinc outflows were lower than the values that cause growth reductions in *L*. *minor* between 0.5 and 15 mg/l (Khellaf and Zerdaoui 2009; Yaseen and Scholz 2017). The standard threshold of zinc for irrigation is 2 mg/l (Metcalf and Eddy 2003). All outflow values were compliant.

**Fig. 7:**
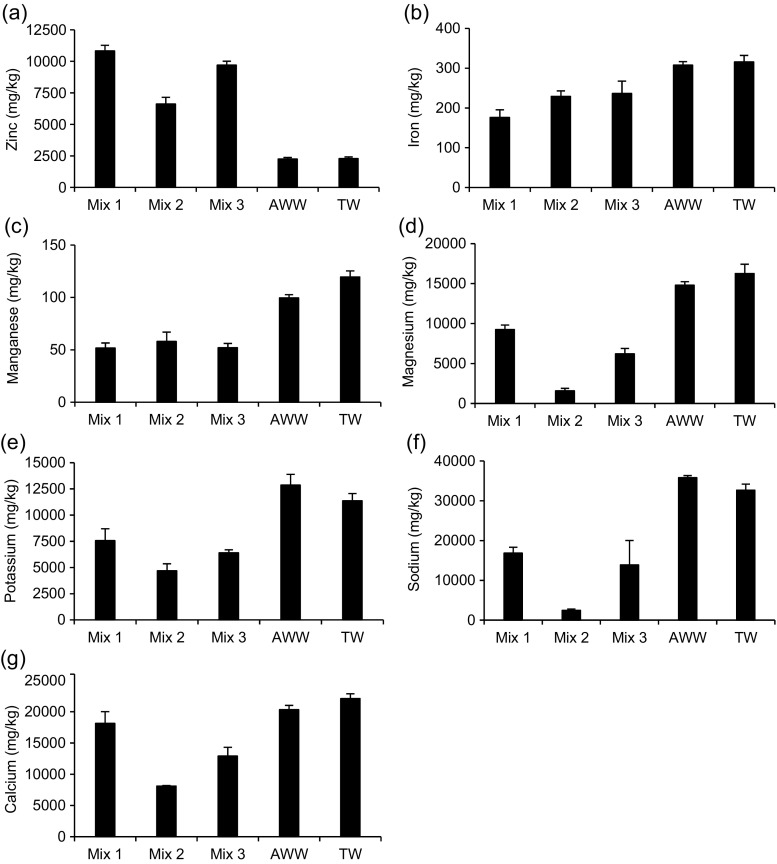
Overview of the means and standard deviations of the concentrations of detected elements in plant tissue. **a** Zinc. **b** Iron. **c** Manganese. **d** Magnesium. **e** Potassium. **f** Sodium. **g** Calcium. Mix1, 8 mg/l of basic red 46 and 2 mg/l of reactive blue 198; Mix2, 2 mg/l of basic red 46 and 8 mg/l of reactive blue 198; Mix3, 5 mg/l of basic red 46 and 5 mg/l of reactive blue 198. AWW artificial wastewater, TW tap water

Regarding iron, the planted ponds showed high outflow concentrations compared to the control ones for all dye mixtures, which can be attributed to plant die-off and subsequent decomposition. *L*. *minor* is able to take-up iron from the water and accumulate this metal in its tissue (see also Fig. 7b). However, when this plant dies during its natural life cycle or due to the toxic impacts of water contaminants, these dead plants consequently will be a source of iron and other elements in the liquid phase. Significant difference between planted and control ponds were found for mixtures one and two (*t* test, *p* < 0.05), which reflect high growth limitations and decompositions related to these ponds. The standard limit of iron for irrigation is 5 mg/l (Metcalf and Eddy 2003).

Concerning manganese (Fig. 2c), inflow concentrations were not detectable. This is because the only source of manganese in the system was tap water. However, outflow values were detectable during the last few months of operation. This is attributed to low manganese reduction and weekly accumulation within the ponds. Outflow concentrations were higher in the control ponds than in the *L*. *minor*-planted ponds, which was ascribed to manganese as a nutrient for plants. All outflow values were less than the maximum allowable concentration for irrigation of 0.2 mg/l (Metcalf and Eddy 2003).

Higher mean outflow concentrations than inflow ones were found for magnesium, sodium and calcium (Fig. 2d–g, respectively) due to the weekly dosages and low reductions except for potassium (Fig. 2e) in all mixtures. The ponds not exposed to the dyes showed lower outflows related to these elements than ponds containing dye, because the plants were healthier and their ability for growth and acquirement of micro-nutrients was higher.

Figure 7 provides an overview of the concentrations of elements accumulated in plant tissues for all ponds with and without dyes. The plants linked to ponds without dye showed higher capacity for element accumulation due to their growth state, except for zinc, which could be due to the low inflow zinc concentration in ponds without dyes. The levels of zinc and iron in plants (Fig. 7a, b) were more than the allowable boundaries of 50 and 20 mg/kg, respectively, as mentioned by Nazir et al. (2015).

**Fig. 8:**
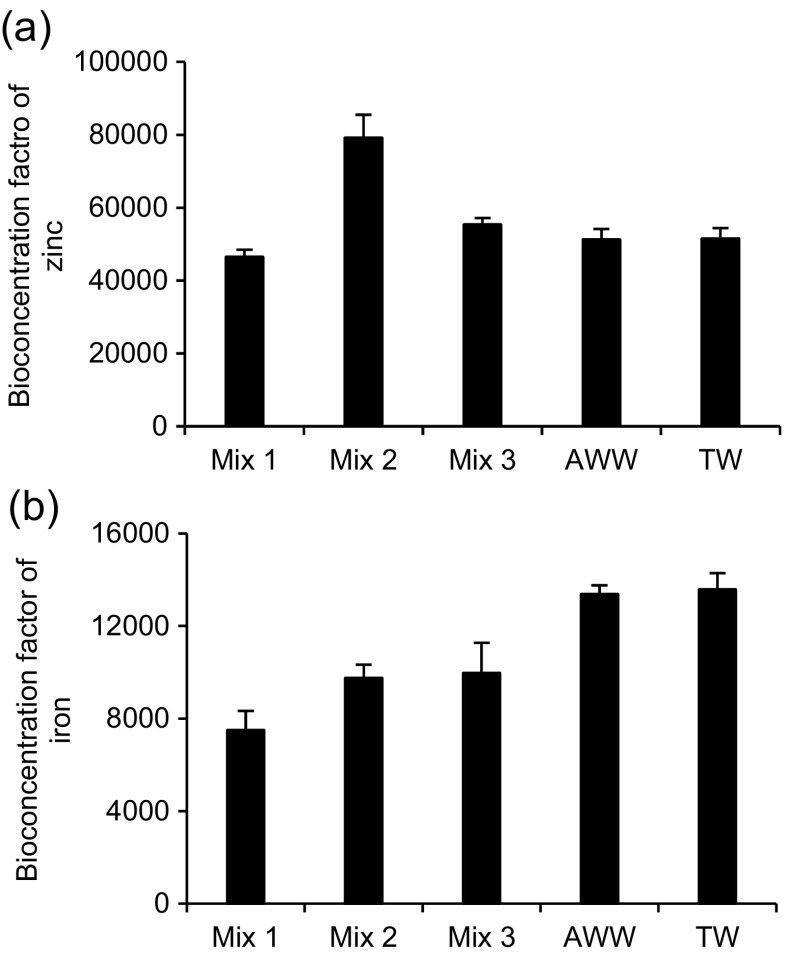
Bio-concentration factor of the main heavy metals detected through the inductively coupled plasma optical emission spectrometer analysis. **a** Zinc. **b** Iron. Mix 1, 8 mg/l of basic red 46 and 2 mg/l of reactive blue 198; Mix 2, 2 mg/l of basic red 46 and 8 mg/l of reactive blue 198; Mix 3, 5 mg/l of basic red 46 and 5 mg/l of reactive blue 198. AWW artificial wastewater, TW tap water. Note that the error bars relate to two replicates of plants

Bio-concentration factors (Fig. 8) are indicators of the potential of the plants for accumulating of heavy metals. A bio-concentration factor of higher than 1000 indicates that plants can positively accumulate heavy metals as mentioned by Sukumaran (2013). The results in Fig. 8a, b indicate that plants were positive for phytoremediation of zinc and iron, respectively.

#### Other water quality parameters

Regarding pH, all inflow and outflow (Tables 3 and 4, respectively) values were within the tolerable range for *L*. *minor* growth, which is between 4.0 and 9.0, as mentioned by Movafeghi et al. (2013) and for bacteria survival between 4.0 and 9.5 (Kadlec and Wallace 2009). The mean pH outflow was slightly more than the mean inflow for all ponds, and all values were within the neutral range. These results confirmed that values of pH were within the optimum range, between 6 and 10, for high colour elimination (Saratale et al. 2011), although the exact amount of pH for higher removal is dependent on the dye itself (Yaseen and Scholz 2016). No significant (*p* > 0.05) differences were found between vegetated and control ponds. The international lower and upper thresholds for pH are 6.5 and 8.5 for safe discharge to receiving watercourses. All outflow values were compliant.

The redox potential is used as an indicator for the presence of aerobic (≥ 100 mV) or anaerobic (≥ 100 mV) environments in wetlands (Ong et al. 2009). Generally, minimum and maximum redox potential outflow values for all ponds were between − 80.5 and − 36.5 mV, respectively, which indicates that anoxic conditions dominate.

Based on DO, all ponds with and without dyes showed lower outflow concentrations compared with the inflow ones (Tables 3 and 4, respectively). The outflow concentrations were high and varied between 8.0 and 8.9 mg/l. No significant differences (*t* test, *p* > 0.05) were found between *L*. *minor* and control ponds in terms of mean outflow DO values. These results indicate that the main source of oxygen in the system was atmospheric diffusion, and the plants did not play a major role in enhancing the DO content.

Concerning SS, all ponds with and without dyes showed high mean SS outflow values (Table 4), if compared with the inflow concentrations (Table 3). This reflects that the COD and other organic substances degrade in addition to organic dye molecule degradation in case of ponds containing dyes. Also, planted ponds showed that outflows were significantly (Mann-Whitney U, *p* < 0.05) higher than the corresponding concentrations for control ones. This can probably be attributed to the impact of *L*. *minor* and their die-off, which enhanced the organic load (Dalu and Ndamba 2003) as well as the effect of higher COD removal, molecule degradation and aromatic amine mineralisation in planted ponds compared with unplanted ones. A typical international standard limit for SS is 35 mg/l; all outflow values of SS were lower than this limit.

The mean inflow and outflow turbidity values (Tables 3 and 4) had the same trend as SS, and the planted ponds also showed an outflow turbidity significantly higher (Mann-Whitney U, *p* < 0.05) than the unplanted ones. Note that correlation analysis showed that SS was significant (*p* < 0.01) and correlated positively (*r* = 0.281, *p* = 0.000) with turbidity.

Based on EC, all mean outflow numbers were a bit higher than the corresponding influent values. Planted ponds showed lower EC values than the control ones, which confirms that *L*. *minor* has a good ability in reducing the EC. Similar findings were published by Yassen and Scholz (2016), who have treated different dyes individually. However, the differences were significant (Mann-Whitney U, *p* < 0.05) in terms of *L*. *minor* compared with control ponds regarding EC outflows founded in case of mixtures two and three. This was perhaps due to the state of *L*. *minor* in the system, plants being healthier in ponds treating mixture two followed by mixture three, if compared with the plants within mixture one.

Regarding TDS values, the inflow and outflow concentrations of TDS in each mixture were a mirror to the corresponding inflow and outflow EC values. This is because TDS concentrations are a function of EC. In this study, TDS values were equal to half of the EC values as mentioned earlier by Yaseen and Scholz (2017). According to common international standards, the results showed that all outflow TDS concentrations were less than the threshold of 500 mg/l.

#### Environmental conditions

The optimum temperature required for ideal growth of *L*. *minor* has been reported by Ozengin and Elmaci (2007) as 26 °C. *L*. *minor* growth reduction occurs at temperature values below 17 °C and higher than 35 °C. In this study, laboratory conditions were controlled and the mean temperature value was 23 °C. The maximum and minimum values were 27 and 19 °C, respectively (Table 3). These results indicate that temperature records in this study do not have any adverse impact on the growth of *L*. *minor* in the system, although the growth will not be at an optimum rate.

The mean record of light intensity was 6853 lx. The corresponding maximum and minimum readings were 7722 and 6335 lx in that order (Table 3). These values were within the suitable range, between 1480 lx and 8140 lx, for high rate of *L*. *minor* production as mentioned by Yin et al. (2015).

#### Plant monitoring

The growth of *L*. *minor* was monitored during the experimental period between 14 October 2016 and 27 June 2017 as an indicator for the toxic impact of each dye mixture as well as the artificial wastewater. During the first 2 months of the experimental operation, the growth of *L*. *minor* in all mixtures was limited. This period can be seen as an acclimatisation stage for plants with dye mixtures that contained two dyes with a total concentration of 10 mg/l and artificial wastewater chemicals.

After the set-up phase, the plants started to increase in numbers and cover some of the surface area of the ponds. Although, a full coverage of the simulated pond surface was not achieved. After March 2017, the toxic signs of the dye mixture were obvious in most systems; green fronds of *L*. *minor* turned light green and yellow. Although the plants associated with mixture three looked healthier and better than those in mixture one during the experiment, most *L*. *minor* plants in both mixtures were dead (partly dry at the sides of the pond walls) when the experiment was finished. The remaining plants turned dark brown, and no obvious growth was noticed.

Most plants within mixture two were light green (2.5 GY; Munsell Color 1977). The remaining plants were white. The growth rate was also inhibited in ponds comprising mixture two in comparison to ponds without dyes. Ponds comprising artificial wastewater and tap water showed that most of the leaves of *L*. *minor* were light green (2.5 GY) and dark green (7GY) according to Munsell Color (1977). Plants within artificial wastewater were mostly green and healthier than those in tap water due to the presence of sufficient nutrients in the artificial wastewater. The growth rate of *L*. *minor* (Table 5) was as follows: ponds containing tap water > ponds containing artificial wastewater > ponds containing mixture two.

**Table 5:** Overview of *Lemna minor* L. (four replicate) findings during the experimental period between 14 October 2016 and 27 June 2017

Treatment system	Fresh weight (g)	Dry weight (g)	Relative growth rate per day
Mixture 1	2.150 ± 0.1872	0.102 ± 0.0165	N/A
Mixture 2	5.793 ± 1.1150	0.309 ± 0.0703	0.00425 ± 0.001517
Mixture 3	2.250 ± 0.1118	0.103 ± 0.0077	N/A
Artificial wastewater	10.241 ± 0.4101	0.609 ± 0.0238	0.00793 ± 0.000209
Tap water	10.437 ± 0.8689	0.622 ± 0.04344	0.00801 ± 0.000433

Mixture 1 (8 mg/l of basic red 46 + 2 mg/l reactive blue 198), mixture 2 (2 mg/l of basic red 46 + 8 mg/l reactive blue 198), mixture 3 (5 mg/l of basic red 46 + 5 mg/l reactive blue 198); each mixture mixed with artificial wastewater

*N/A* not applicable

Significant (Kruskal–Wallis , *p* < 0.05) lower growth was noted for ponds treating mixture two compared to ponds containing tap water and artificial wastewater. These results suggest that a mixture of dyes restrains the photosynthesis process, impacts on the chlorophyll pigments in the fronds and consequently inhibits the growth of the plants. Particularly, when the concentration of the dye BR46 in the mixture was high and the dye removal was at a maximum.

Findings regarding chlorophyll pigments do not agree with some previous publications (Yaseen and Scholz 2016; Khataee et al. 2012), where the authors proved that the dyes do not adversely affect the chlorophyll content, although the observed growth rate of *L*. *minor* was reduced. This can be attributed to the separate dye preparation process in aqueous solution (Khataee et al. 2012) and the benefits of fertiliser application (Yaseen and Scholz 2016) compared to the mixture of dyes added to artificial wastewater chemicals in this research. However, the results of the plant growth rate in each mixture matched the findings published by Movafeghi et al. (2013). The authors found that an increase in BR46 concentration to 10 mg/l was linked to a reduction in *L*. *minor* growth rate, although the inhibition in this study was higher at the presence of this dye at concentrations < 10 mg/l, which were 8 mg/l in the first mixture followed by the third and second mixture of 5 and 2 mg/l, respectively. Therefore, a growth reduction of *L*. *minor* was due to the presence of three adverse factors working together in combination: dye mixture, concentration of BR46 and artificial wastewater.

Furthermore, zinc was also present within BR46, although it was within the tolerable range for plant growth within the outflow samples. However, the concentration of zinc accumulated within plant tissue was higher than the allowable limit as clarified earlier. This could be the main reason for plant damage in ponds containing BR46. The impact of zinc treated by *L*. *minor* was investigated by Radic et al. (2010). The authors concluded that zinc caused a reduction in plant growth and chlorophyll pigments.

This study also showed that the artificial wastewater had a slightly negative influence on plant growth, which could be negligible, if compared with plants in tap water. Significantly (Kruskal–Wallis , *p* > 0.05), no differences were observed between ponds containing artificial wastewater and tap water regarding *L*. *minor* growth. However, the growth of *L*. *minor* within tap water ponds (Table 5) was significantly lower in comparison with the same species of plants subjected to TNC complete fertiliser for optimum growth, which led to a growth rate of around 0.011 per day (Yaseen et al. 2017). This indicates that the growth of *L*. *minor* in ponds containing tap water and artificial wastewater inhibited plant growth.

Within the last period of the experiment, an algal biofilm appeared in the bed of each pond (including those without dyes), which was due to the low growth rate of *L*. *minor* providing an uncovered surface area. However, the impact of algae was neglected during their appearance within the last weeks of the experimental operation, because the results were stable.

## Conclusions

This study concluded that simulated pond systems (microcosms) effectively improved the main parameters of water quality including COD, NH_4_-N and NO_3_-N, but not PO_4_-P. A considerable impact of *L*. *minor* ponds compared with the control ones was noted. The outflow values of pH, COD, NH_4_-N, NO_3_-N, SS and TDS were within the acceptable limits for direct discharge. The planted pond efficiency in terms of removing dye mixtures was significantly (*p* < 0.05) higher than that for unplanted ones. High removals were associated with mixtures containing higher percentages of biodegradable dyes.

Colourless outflows were linked to ponds treating 8 mg/l of BR46 mixed with 2 mg/l of RB198. The HPLC and UV–Vis analyses confirmed phyto-transformation and adsorption of BR46 in planted ponds. In addition, GC-MS data confirmed the complete aromatic amine mineralisation for the treated dye BR46 within the mixtures to water and carbon dioxide in planted ponds, whereas the control ponds showed the presence of N-(4-methylphenyl)-benzenemethanamine in the outflow samples.

The artificial wastewater reduced the growth of *L*. *minor*. However, dye mixtures have a toxic impact on *L*. *minor*, particularly when the concentration of BR46 in the mixture was 5 mg/l or more. This study also concluded that the treatment of BR46 as part of mixed dyes is worse concerning its degradation potential compared to the corresponding individual dye solution. Overall, this study suggests that *L*. *minor* ponds are more effective to operate in case of wastewater treatment, containing separate (or individual) textile dyes, although the pond systems are able to treat mixtures of dyes.
